# Supplementary material for: Green Tea Epigallocatechin 3-Gallate Reduced Platelet Aggregation and Improved Anticoagulant Proteins in Patients with Transfusion-Dependent β-Thalassemia: A Randomized Placebo-Controlled Clinical Trial
Source: Foods. 2024 Nov 29;13(23):3864. doi: 10.3390/foods13233864 (PMC11640449; doi:10.3390/foods13233864)
Supplement: Supplementary file 1 [file foods-13-03864-s001.zip › foods-3245319-supplementary.pdf]

Supplementary Methods

S1. Preparation of Green Tea Extract

Briefly, fresh tea (*Camellia sinensis*) shoots were harvested from tea fields of the Royal Project Foundation at Mon-ngao, Mae Taeng, Chiang Mai Province and immediately dried at 100 °C for 3 minutes in an electric microwave oven (Electrolux, Stockholm, Sweden, 20−L capacity, 4000−watts, 220 volts electric power) to inactivate inherent polyphenol oxidase. Dry tea leaves were finely ground with an electric blender (SharpThai Company, Limited, Thailand) and the powder (1 kg) was extracted in 10 L of hot deionized water (DI) at 80 °C for 10 minutes and ultrafiltrated through a filter membrane (cellulose acetate type, 50 mm diameter, 0.45 μm pore size, GE Healthcare Life Sciences, Whatman, Maidstone, Kent, UK) under a vacuum [1]. Finally, green tea extract (GTE) solution was dried under a condition of -60 °C, <5 Pa with a vacuum freeze-drying machine (4-kg capacity, Brand: ZZKD, Manufacturer number: 0606267299562, Manteste Equipment Company, Zheng zhou, China).

S2. Production of GTE and Placebo Tablets

The liquid GTE was mixed with maltodextrin (5%, w/v) and rapidly dried using a spray dryer to transform the liquid into fine GTE particles. Afterward, the spray-dried GTE powder was mixed with microcrystalline cellulose (MCC) (2:1, w/w) and polyvinylpyrrolidone-K90 (PVPK90) previously dissolved in 95% ethanol at a final concentration of 1% (w/w), filtered through a 14-mesh nylon net filter, and dried using a fluid-bed dryer. Finally, the GTE granules were compressed into an oval-shaped tablet existing 50 mg epigallocatechin 3-gallate (EGCG) each [2]. Placebo tablets matched to the size, shape, and color of the GTE tablets were produced similarly which excipients of maltodextrin and food colorizing agents, except for active ingredient spray-dried GTE powder, were mixed homogenously and processed as described before. Then, GTE and placebo tablets were kept in white polypropylene bottles (30 tablets each) with sealed caps at 4 °C in a refrigerator until being used.

S3. HPLC/DAD Analysis of Catechin Derivatives

Catechin derivatives were quantified using the high-performance liquid chromatography/diode array detector (HPLC/DAD) method [2]. First of all, a GTE tablet was ground in a ceramic cup, consequently the tablet powder (10 mg) was reconstituted in of DI water (1.0 mL) and filtered through a syringed polyvinylidene fluoride (PVDF) membrane filter (0.45-μm pore size, Monotaro Company, Limited, Tokyo, Japan). In analysis, 20 μL of sample or standard catechins including catechin (C), epicatechin (EC), epicatechin 3-gallate (ECG) and epigallocatechin 3-gallate (EGCG) (1 mg/mL each) were loaded into the HPLC coupled with a DAD, fractionated onto a column (C18-type, 150 mm x 4.6 mm, 5 μm, Agilent Technologies, Inc., Santa Clara, CA, USA), eluted with mobile-phase solvent of 0.05% H_2SO_4: acetonitrile: ethyl acetate (86:12:2 by volume) at a flow rate of 1.0 mL/minute and the eluents were then detected at 280 nm. The catechins existing in the samples were identified using the same retention time (T_R) as the authentic standards and determined the concentrations using their standard curves.

S4. HPLC/DAD Analysis of Caffeine

Caffeine (CF) contents were determined in both the GTE and placebo tablet using the HPLC/DAD method [3]. A stock standard CF solution (500 μg/mL) was firstly prepared in DI water, and a working CF solution (50 μg/mL) was subsequently prepared by diluting the stock solution with DI water. Placebo and GTE tablets (1.0 g each) were accurately weighed, crushed in a ceramic cup, dissolved in hot DI water (about 90 °C), placed in a boiling water bath for about 30 minutes, cooled down, and then transferred to a 50-mL volumetric flask. The solution was then filtered through the syringe PVDF membrane filter before being used. The resulting sample (20 μL) was injected into the HPLC/DAD machine and fractionated on a column (μBondapack C18 type, 3.9 mm x 300 mm, 10 μm, Waters Corporation, Milford, MA, USA) capped with an equivalent guard column and eluted with the mobile-phase solvent (DI water/methanol/acetic acid = 80/19/1 by volume) at a flow rate of 1.5 mL/minute. Eluents were then detected at 276

nm. Concentrations of CF were determined using the standard CF calculation and converted to the appropriate content in the tablet.

S5. HPLC/DAD Analysis of Ascorbic Acid

Vitamin C or ascorbic acid (AA) content was analyzed using an HPLC/DAD method [4]. Placebo and GTE tablets were crushed finely, dissolved in 3% (w/v) m-phosphoric acid (MPA), shaken vigorously for 2 minutes, sonicated in an ultrasonic bath for 5 minutes, and made up to 100 mL with MPA. Standard AA solution (10 mg/100 mL) was freshly prepared by dissolving 10 mg of AA in 3% MPA, homogenizing it with a mixer, and making up to 100 mL with MPA in a volumetric flask. The solutions were then filtered through the syringe PVDF type, 0.45 µm membrane filter before being used. The resulting samples (20 µL) were injected into the HPLC/DAD machine and fractionated on a column (Lichrocard Lichrosphere 100 RT C18 type, 3.5 mm x 125 mm, 5 µm, Merck KGaA, Darmstadt, Germany) capped with an equivalent guard column and eluted at ambient temperature with 3 mM potassium dihydrogen phosphate in 0.35% (v/v) o-phosphoric acid pH 3.0 as the mobile-phase solvent at a flow rate of 0.5 mL/minute, and detected the eluents at 244 nm. The peak and concentration of AA in the GTE and placebo samples were positioned to that of standard AA and calculated to the appropriate content in the tablet.

S6. HPLC/FLD Analysis of Tocopherols and Tocotrienols

Contents of tocopherols and tocotrienols were determined in both the placebo and GTE tablet using the normal-phase HPLC/fluorescence detection (FLD) method [5]. A standard mixture solution was comprised of α-tocopherol (91.65 µg/mL), α-tocotrienol (84.49 µg/mL), β-tocopherol (39.40 µg/mL), γ-tocopherol (76.0 µg/mL), β-tocotrienol (24.85 µg/mL), γ-tocotrienol (90.81 µg/mL), δ-tocopherol (82.53 µg/mL), and δ-tocotrienol (76.04 µg/mL). A sample (placebo and GTE tablet) was ground and saponified under nitrogen in screw capped tubes with 2.0 mL of potassium hydroxide (60 g/dL), 2.0 mL of 95% ethanol, 2.0 mL of sodium chloride (1 g/dL), and 5.0 mL of ethanolic pyrogallol (6 g/dL) as an antioxidant in a 70 °C water bath for 45 minutes. After alkaline digestion, we allowed the sample to cool down. Subsequently, 15 mL of the sodium chloride solution was added to the tubes and the suspension was extracted twice with a 15 mL portion of n-hexane/ethyl acetate (9:1 by volume). The organic layer was collected and dried using a speed-vac centrifuge, while the dry residue was dissolved in 2.0 mL of isopropanol (1%) in n-hexane. In our analysis, the sample and standard (50 µL) solutions were injected automatically into the HPLC machine (Agilent Technologies 1100 Series, Deutschland GmbH, Waldbronn, Germany) consisting of a quaternary pump (G1311A), an online vacuum degasser (G1322A), and an autosampler (G1313A). They were then fractionated on a column (Kromasil Phenomenex Si column, 150 mm x 4.6 mm, 5 µm, Torrance, CA, USA) that was regulated thermally at 40 °C with a column compartment regulator (G1316A), eluted with the mobile–phase solvent (n-hexane/ethyl acetate/acetic acid = 97.3/1.8/0.9 by volume) at a flow rate of 1.6 mL/minute, and eluents were detected using a fluorescence detector (Model 1260 Infinity II, Agilent Technologies 1100 Series, Deutschland GmbH, Waldbronn, Germany) at 270 nm and an excitation at 330 nm. Concentrations of individual tocopherols and tocotrienols were determined using calculations of authentic standards. They were then converted into the appropriate content in the tablet.

Supplementary Figures

A

B

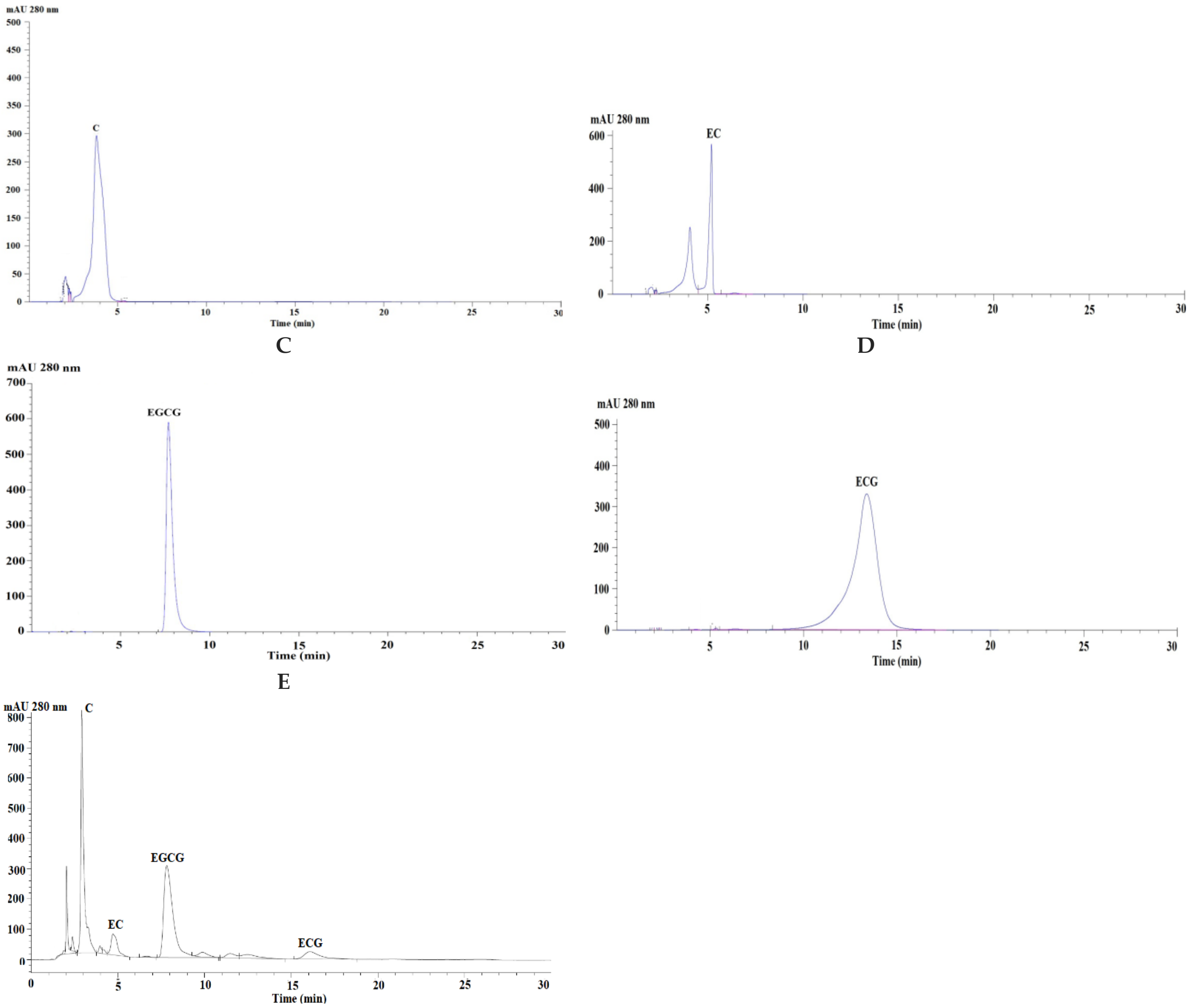

Supplementary Figure 1. HPLC/DAD profiles of standard C (A), EC (B), EGCG (C) and ECG (D), and catechins in GTE (0.1 mg/mL each) (E). Abbreviation: C = catechin, EC = epicatechin, ECG = epicatechin 3-gallate, EGCG = epigallocatechin 3-gallate, HPLC/DAD = high performance liquid chromatography/diode array detection, mAU = milli-absorbance unit.

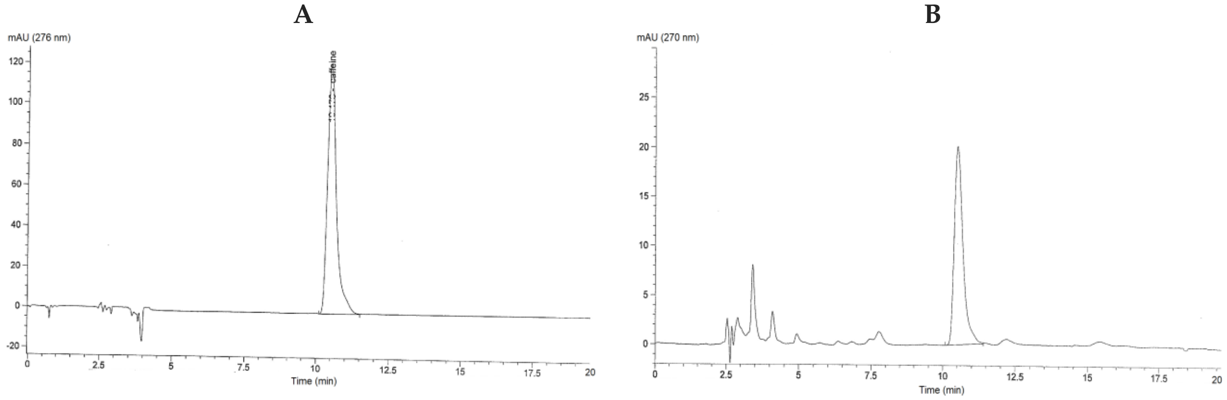

Supplementary Figure 2. HPLC/DAD profiles of caffeine in authentic standard (A) and GTE (B) (1 mg/mL each). Abbreviation: HPLC/DAD = high performance liquid chromatography/diode array detection, mAU = milli-absorbance unit.

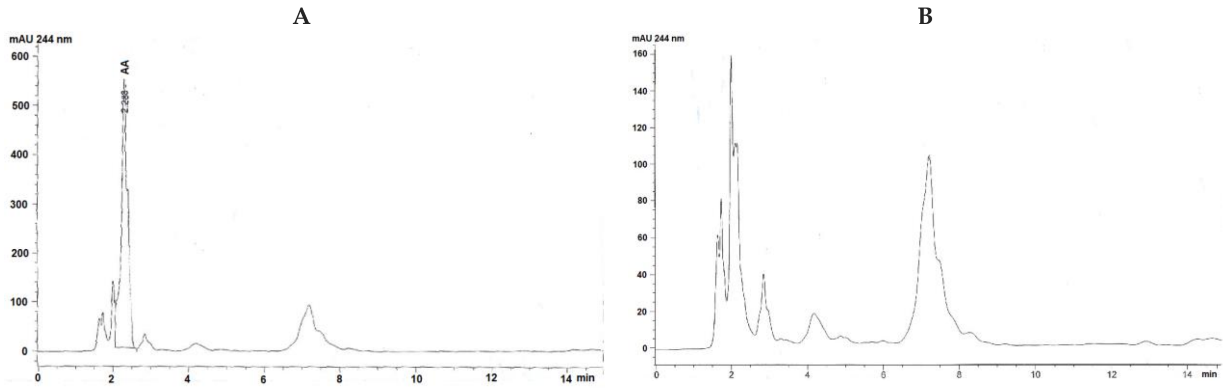

Supplementary Figure 3. HPLC/DAD analysis of AA in authentic standard (A) and GTE (B) (10 mg/mL each). Abbreviation: AA = ascorbic acid, HPLC/DAD = high performance liquid chromatography/diode array detection, mAU = milli-absorbance unit.

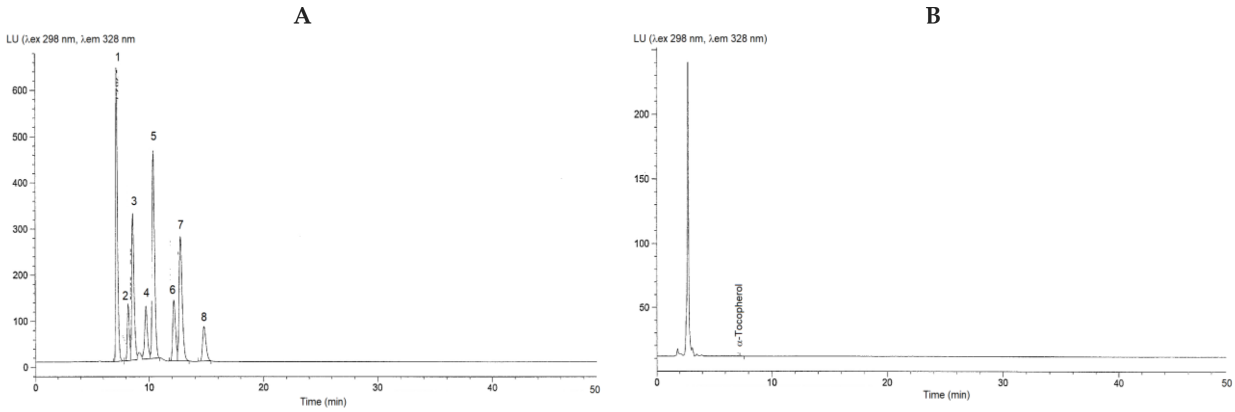

Supplementary Figure 4. HPLC/FLD profiles of tocopherols and tocotrienols in authentic standards (A) and GTE tablet (B) (10 mg/mL). Abbreviation: AA = ascorbic acid, HPLC/FLD = high performance liquid chromatography/fluorescence detection, LU = luminescence unit.

Supplementary Tables

Supplementary Table 1. Ingredient compositions in GTE granule (1 g), a placebo and GTE tablet (size 750 mg each).

Constituents	GTE granules	Placebo tablet	GTE tablet
Catechins			
C (mg)	98.27	ND	98.27
EC (mg)	18.11	ND	18.11
ECG (mg)	49.99	ND	49.99
EGCG (mg)	0.59	ND	0.59
CF (mg)	9.8	ND	0.74
α-Tocopherol (μg)	0.04	ND	0.045
Maltodextrin (mg)	-	24.75	24.75
Food colorizing agents (mg)	-	470.25	-
MCC (mg)	-	247	247
PVP-K90 (mg)	-	7.42	7.42

Abbreviations: C = catechin, CF = caffeine, EC = epicatechin, ECG = epicatechin, EGCG = epigallocatechin 3-gallate, MCC = microcrystalline cellulose, ND = not detected, PVPK90 = polyvinylpyrrolidone-K90.

Supplementary Table 2. Information and some blood biomarker levels of normal and thalassemia subjects at the time of blood collection.

Subject	Dx	Sex	Age (y)	BW (kg)	Ht (cm)	BMI (kg/m^2)	Iron chelator	ASA	SPX	Liver spam (cm)	PLT (x10^5 cells/mm^3)	Ft (ng/mL)
N1	N	F	25	53	150	23.6	No	No	No	ND	2.11	96
N2	N	F	27	51	157	20.7	No	No	No	ND	1.96	104
N3	N	F	28	64	165	23.5	No	No	No	ND	2.34	103
N4	N	M	27	68	165	25.0	No	No	No	ND	2.66	87
N5	N	M	27	70	173	23.4	No	No	No	ND	2.45	111
N6	N	M	27	72	170	24.9	No	No	No	ND	2.63	92
NTDT	BE	F	46	56	160	35.0	DFP	No	No	10	2.97	849
TDT1	BM	F	33	45	139	32.4	DFP	Yes	Yes	10	5.75	1016
TDT2	BM	M	36	57	167	34.1	DFP	Yes	Yes	13	4.55	783
TDT3	BE	M	28	44	160	27.5	DFX	Yes	Yes	13	10.35	2154
TDT4	BM	F	35	57	156	36.5	DFP	Yes	Yes	12	6.31	689
TDT5	BE	M	20	62	180	34.4	DFP	No	No	10	1.72	1064
TDT6	BM	M	25	54	160	33.8	DFP	Yes	Yes	16	5.56	751
TDT7	BM	F	36	48	149	32.2	DFO+DFX	Yes	Yes	12	2.63	1680
TDT8	BM	M	30	43	160	26.9	DFP	Yes	Yes	12	4.64	943
TDT9	BM	F	37	60	165	36.4	DFP+DFO	No	No	13	9.11	1050

Abbreviation: ASA = acetylsalicylic acid, BE = β-thalassemia/hemoglobin E, BM = β-thalassemia major, BMI = body mass index, BW = body weight, DFO = desferrioxamine, Dx = diagnosis, DFX = deferasirox, F = female, Ft = serum ferritin, GPO-L-ONE = deferiprone, GTE = green tea extract, H = hemoglobin H disease, M = male, N = normal, NA = not available, ND = not done, NTDT = non-transfusion-dependent thalassemia, PLT = platelet, SD = standard deviation, SPX = splenectomy, TDT = transfusion-dependent thalassemia.

References

1. Koonyosying, P., Kongkarnka, S., Uthaipibull, C., Svasti, S., Fucharoen, S., and Srichairatanakool, S. Green tea extract modulates oxidative tissue injury in beta-thalassemic mice by chelation of redox iron and inhibition of lipid peroxidation. Biomed Pharmacother. 2018 Dec; 108, 1694-1702. 10.1016/j.biopha.2018.10.017.
2. Settakorn, K., Hantrakool, S., Petiwathayakorn, T., Hutachok, N., Tantiworawit, A., Charoenkwan, P., Chalortham, N., Chompupoung, A., Paradee, N., Koonyosying, P., and Srichairatanakool, S. A randomized placebo-controlled clinical trial of oral green tea epigallocatechin 3-gallate on erythropoiesis and oxidative stress in transfusion-dependent beta-thalassemia patients. Front Mol Biosci. 2023 10, 1248742. 10.3389/fmolb.2023.1248742.
3. Luca, V.S., Stan, A.M., Trifan, A., Miron, A., and Aprotosoaie, A.C. Catechins Profile, Caffeine Content and Antioxidant Activity of Camellia Sinensis Teas Commercialized in Romania. Rev Med Chir Soc Med Nat Iasi. 2016 Apr-Jun; 120, 457-463.
4. Uckoo, R.M., Jayaprakasha, G.K., Nelson, S.D., and Patil, B.S. Rapid simultaneous determination of amines and organic acids in citrus using high-performance liquid chromatography. Talanta. 2011 Jan 15; 83, 948-954. 10.1016/j.talanta.2010.10.063.
5. Panfili, G., Fratianni, A., and Irano, M. Normal phase high-performance liquid chromatography method for the determination of tocopherols and tocotrienols in cereals. J Agric Food Chem. 2003 Jul 2; 51, 3940-3944. 10.1021/jf030009v.
